# Discrete-time quantum walk with feed-forward quantum coin

**DOI:** 10.1038/srep04427

**Published:** 2014-03-21

**Authors:** Yutaka Shikano, Tatsuaki Wada, Junsei Horikawa

**Affiliations:** 1 Research Center of Integrative Molecular Systems (CIMoS), Institute for Molecular Science, Okazaki, Aichi 444-8585, Japan; 2 Institute for Quantum Studies, Chapman University, Orange, California 92866, USA; 3 Department of Electrical and Electronic Engineering, Ibaraki University, Hitachi, Ibaraki 316-8511, Japan; 4 Major in Materials Science, Graduate School of Science and Engineering, Ibaraki University, Hitachi, Ibaraki 316-8511, Japan

## Abstract

Constructing a discrete model like a cellular automaton is a powerful method for understanding various dynamical systems. However, the relationship between the discrete model and its continuous analogue is, in general, nontrivial. As a quantum-mechanical cellular automaton, a discrete-time quantum walk is defined to include various quantum dynamical behavior. Here we generalize a discrete-time quantum walk on a line into the feed-forward quantum coin model, which depends on the coin state of the previous step. We show that our proposed model has an anomalous slow diffusion characterized by the porous-medium equation, while the conventional discrete-time quantum walk model shows ballistic transport.

Cellular automata – discrete models that follow a set of rules^1^ – have been analyzed in various dynamical systems in physics, as well as in computational models and theoretical biology; well-known examples include crystal growth and the Belousov-Zhabotinsky reaction. To simulate quantum mechanical phenomena, Feynman^2^ proposed a quantum cellular automaton (the Feynman checkerboard). This model, defined in the general case by Meyer^3^, is known as the discrete-time quantum walk (DTQW). Since the DTQW on a graph is a model of a universal quantum computation^4^,^5^, it is of great utility, especially in quantum information^6^,^7^,^8^,^9^. Furthermore, the DTQW has been demonstrated experimentally in various physical systems^10^,^11^,^12^,^13^,^14^,^15^,^16^,^17^,^18^,^19^,^20^,^21^,^22^,^23^,^24^ to reveal quantum nature under dynamical systems.

As the cellular automaton can be mapped to various differential equations by taking the continuous limit, some DTQW models can be mapped to the Dirac equation^25^,^26^,^27^, the spatially discretized Schrödinger equation^28^,^29^, the Klein-Gordon equation^27^,^30^, or various other differential equations^31^,^32^. These equations have ballistic transport properties, which are reflected mathematically in the one-dimensional (1D) DTQW with a time- and spatial-independent coin operator, i.e. a 1D *homogeneous* DTQW^33^. We consider here the 1D DTQW model. Physically, the standard deviation of the homogeneous DTQW is *σ*(*t*) ~ *t*, whereas the unbiased classical random walk has a standard deviation of 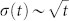.

In the homogeneous DTQW, the time evolution of a quantum particle (walker) is given by a unitary operator *U* defined on the composite Hilbert space 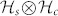, where 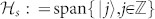 is the walker Hilbert space, and 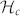 is the two-dimensional coin Hilbert space. For a unitary operator *U*, the quantum state evolves in each time step *t* by 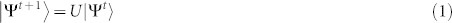
with 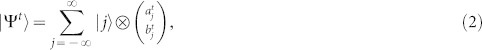
where the upper 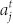 (lower 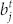) component corresponds to the left (right) coin state at the *j*-th site at time step *t*. As an example, the time evolution of the DTQW is given by 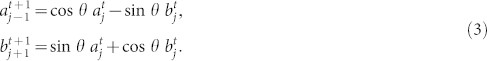
The *j*-th site probability at time step *t* is given by 
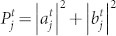
, and 
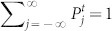
 is satisfied for each time step *t*.

As a generalization of Eq. (3), we define a DTQW with a feed-forward quantum coin described by 
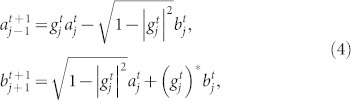
with the site-dependent rate function 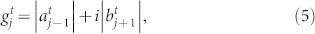
which incorporates the nearest-neighbor interactions. Since this quantum coin depends on the probability distribution of the coin states on the nearest-neighbor sites at the previous step, this model is called a *feed-forward DTQW*. It is remarked that the feed-forward DTQW is one of the nonlinear DTQW models. Note that if we set the rate function 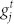 to *g* = cos *θ*, which is time and site independent, then the model in Eq. (4) reduces to the homogeneous model in Eq. (3). We will show that our proposed feed-forward DTQW is experimentally feasible. Furthermore, we will show that this model shows the anomalous diffusion as introduced below.

One of the famous anomalous diffusion equations is the porous medium equation (PME)^34^, defined by 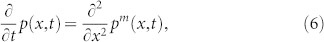
where the real parameter *m* > 1 characterizes the degree of porosity of the porous medium. It is known that the PME can be derived from three physical equations for the density *ρ*, pressure *p*, and velocity **v** of the gas flow: the equation of continuity, ∂*ρ*/∂*t* + ∇ · (*ρ***v**) = 0; Darcy's law, **v** ∝ −∇*p*; and the equation of state for a polytropic gas, *p* ∝ *ρ^ν^*, where *ν* is the polytropic exponent and *m* = *ν* + 1. One of the peculiar features of the PME is the so-called *finite propagation*, which implies the appearance of a *free boundary* separating the positive region (*p* > 0) from the empty region (*p* = 0).

A well-known solution of the PME is the Barenblatt-Pattle (BP) one^35^; it is self-similar, and its total mass is conserved during evolution. The evolutionary behavior of the BP solution was recently studied in the context of generalized entropies and information geometry^36^. The BP solution can also be expressed by Tsallis' one-real-parameter (*q*) generalization of a Gaussian function, i.e., the *q*-Gaussian^37^. In the case of 1D space, the BP solution is 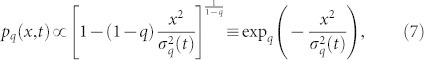
with *q* = 2 − *m*. Here, 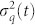 is a positive parameter that characterizes the width of the *q*-Gaussian at time *t* and is similar to the variance 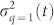 in a standard Gaussian. In other words, the parameter *σ_q_*(*t*) characterizes the spread of the *q*-Gaussian distribution^38^,^39^; 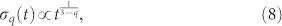
which reduces to 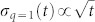 in the limit of *q* → 1. Note that in the same limit, the *q*-Gaussian reduces to the standard Gaussian, 
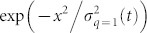
, and the PME reduces to the standard heat equation ∂*p*/∂*t* = ∂^2^*p*/∂*x*^2^.

In this paper, we analyze a specific feed-forward DTQW with an experimental proposal using the polarized state and optical mode. We show numerically that the probability distributions of the feed-forward DTQW model have anomalous diffusion characterized by *σ_q_*_ = 0.5_(*t*) ~ *t*^0.4^. These dynamics are consistent with the time evolution of the self-similar solution^35^ of the PME, which is known to describe well the anomalous diffusion of an isotropic gas through a porous medium. Furthermore, we show analytically that the interference terms in our model help the speedup of the associated Markovian model but does not help the quadratic speedup like the homogeneous DTQW does^40^. Note that although anomalous diffusion was found numerically in a nonlinear model^41^, an aperiodic time-dependent coin model^42^, and the history-dependent coin^43^ from the time dependence of the variance *σ_q_*_ = 1_(*t*), the partial differential equation (PDE) corresponding to their models have not derived due to the lack of the numerical step (about 100 step). Therefore, we have not yet revealed the origin of the anomalous diffusion in the DTQW.

## Results

### Experimental proposal of feed-forward DTQW

We propose an optical implementation of the feed-forward DTQW. In the simple optical implementation of the homogeneous DTQW, the walker space uses the spatial mode and the coin space does the polarized state. The shift uses the polarized beam splitter and the quantum coin uses the quarter-wave, half-wave, and quarter-wave plates, which can arbitrarily rotate the polarized state in the Poincaré sphere. This was experimentally done in Refs. 10,11,12,16,17,18,19,20,21,22.

**Figure 1:**
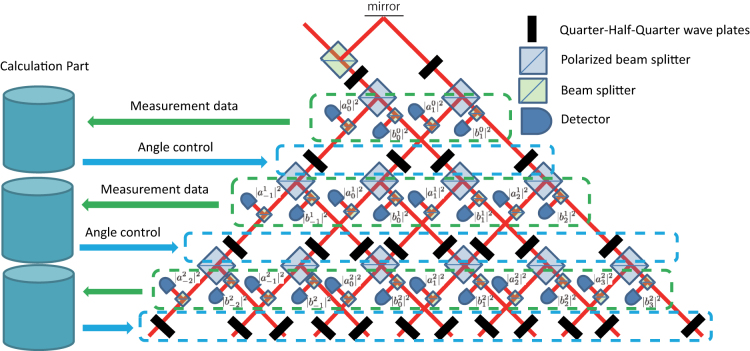
Optical implementation of the feed-forward DTQW model. Figure shows our experimental proposal of our model. From the intensity of the detectors for each path, the polarizers should be changed. This can be taken as the feed-forward quantum coin.

Let us construct the feed-forward system of the quantum coin. The detectors put at each path to evaluate the probability distribution of the coin state 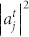 and 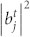. Since our proposed quantum coin depends on 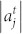 and 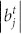, we can calculate the coin operator at the *j*th site. According to the Jones calculation^44^ to satisfy Eq. (4), we control the angels of the quarter-wave, half-wave, and quarter-wave plates for each path. This can be taken as the quantum coin operator with the feed-forward. This is depicted in Fig. 1. In what follows, we consider the long time time evolution of the feed-forward DTQW.

### Numerical results of feed-forward DTQW with anomalous diffusion

To study the time evolution of the feed-forward DTQW model, the initial state should have nonzero coin states at the nearest-neighbor sites. This can be easily understood by considering the following example. Let us take (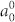, 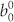) as the only non-zero initial state. In this case, the rate is 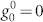, because there is no neighboring state. From the map in Eq. (4), we see that the nonzero states at *t* = 1 are 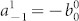 and 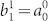. This gives 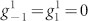, and we see that the only nonzero state is 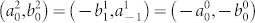 at *t* = 2. This state at *t* = 2 only differs in sign (or phase) from the initial state. Thus if the initial state is concentrated at a single site, no spreading occurs; the state only oscillates around the initial site.

**Figure 2:**
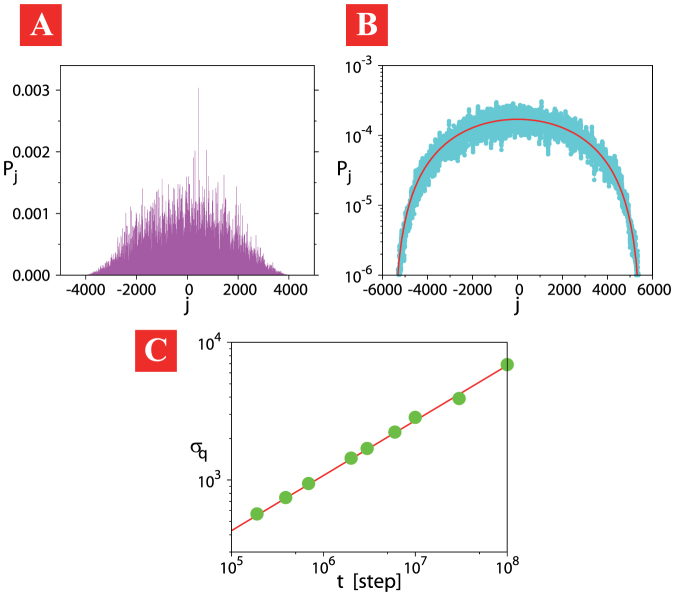
Anomalous slow diffusion of the feed-forward DTQW model. Its probability distribution at *t* = 10^7^ step displayed in Panel (A) with running averaged over 10 data sets (light blue line) is fitted by the *q*-Gaussian (7) with *q* = 0.5 (red line) to obtain the *q*-generalized standard deviation *σ_q_*(*t*) in Panel (B). Panel (C) shows the long-time evolution of the *q*-generalized standard deviation *σ_q_*(*t*) (green dots), which is well fitted by *σ_q_*_ = 0.5_(*t*) ~ *t*^0.4^ (red line).

Figure 2 (A) shows a typical probability distribution of the feed-forward DTQW after a long-time evolution. See the Supplementary Movie for more details. The initial state was set as 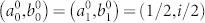. We note that the probability distribution diffuses very slowly and does not approach a Gaussian. These features are often observed in anomalous diffusion. It is also remarked that such behavior has not yet seen in DTQWs with the position-dependent coin^45^,^46^,^47^,^48^, which show the localization property.

We performed long-time numerical simulations of the feed-forward DTQW model [Eq. (4)] for up to *t* ~ 10^8^ steps. To study the asymptotic behavior, we take running averages of the numerical solutions to reduce the influence of multiple spikes. The averaged data were fitted with the *q*-Gaussian of Eq. (7) to determine the corresponding *q*-generalized standard deviation *σ_q_*(*t*), as shown in Fig. 2 (B). We note that the averaged data at each time step are well fitted by the *q*-Gaussian with *q* = 0.5.

The long-time evolution of *σ_q_*(*t*), plotted in Fig. 2 (C), reveals that the time evolution of the feed-forward DTQW model is well characterized by *σ_q_*_ = 0.5_(*t*) ~ *t*^0.4^, which is the same time dependency for *q* = 0.5 of the PME [Eq. (8)].

### Analytical derivation of anomalous diffusion in the associated Markov model of feed-forward DTQW

The relationship between our model and the PME can be explored using the decomposition method of Romanelli *et al.*^40^,^49^, in which the unitary evolution of a DTQW model is decomposed into Markovian and interference terms. We obtain the following map for both coin distributions 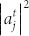 and 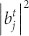: 
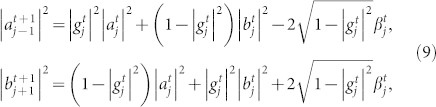
where the two terms including 
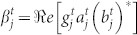
 are interference terms, and 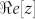 is the real part of a complex number *z*.

**Figure 3:**
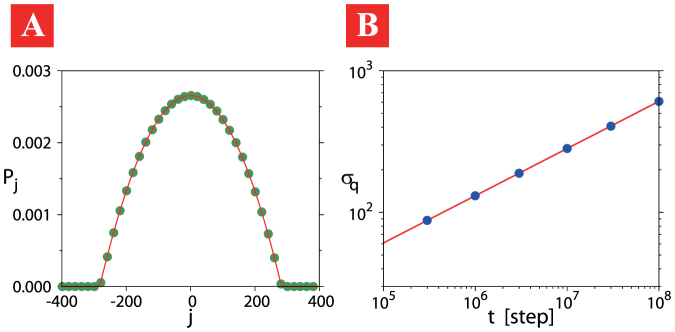
Anomalous slow diffusion of the associated Markovian model for the nonlinear quantum walk. Panel (A) shows the probability distribution of the associated Markovian model at *t* = 10^7^ step (green dots) fitted by the *q*-Gaussian, yielding *q* = 0.0 and *σ_q_*_ = 0.0_(*t*) = 283 (red line). Panel (B) shows the long-time evolution of the *q*-generalized standard deviation *σ_q_*(*t*) of the associated Markovian model (blue dots). It is well fitted by *σ_q_*_ = 0.0_(*t*) ~ *t*^0.33^ (red line).

Neglecting the interference terms and introducing the abbreviations 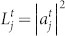 and 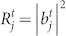, we get the *associated Markovian model*; 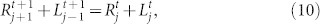


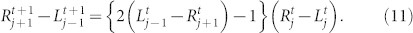
The numerical simulation of the associated Markovian model is performed under initial conditions of 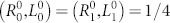, and the typical probability distribution shown in Fig. 3 (A) is well fitted by the *q*-Gaussian with *q* = 0.0. Furthermore, Fig. 3 (B) shows that the time evolution of *σ_q_*(*t*) of the associated Markovian model is well fitted to *σ_q_*_ = 0.0_(*t*) ~ *t*^0.33^, which again is the same time dependency as the PME for *q* = 0.

It is known that the classical Markovian model, i.e. one without the interference terms of the homogeneous DTQW, satisfies the standard heat equation in the continuous limit. Consequently, the associated asymptotic probability distribution is a standard Gaussian. This implies that the ballistic transport property of the homogeneous DTQW comes from the interference term^40^. We thus consider the continuous limit^50^ of the associated Markovian model.

We introduce the density *ρ*(*x*, *t*) and current *j*(*x*, *t*) as 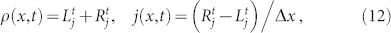
where Δ*x* is the difference of the nearest-neighbor sites. Taking a Taylor expansion of Eq. (10), we get 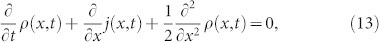
in the diffusion limit, i.e., the quantity (Δ*x*)^2^/Δ*t* remains constant (set to unity here for simplicity) as Δ*t*, Δ*x* → 0 with the one-step time difference Δ*t*. In a similar manner, by expanding Eq. (11) and taking the diffusion limit, we obtain 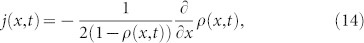
which implies a breakdown in Fick's first law (*j* ∝ −∂*ρ*/∂*x*) and is the hallmark of anomalous diffusion. By substituting Eq. (14) into Eq. (13), we obtain the following nonlinear PDE: 
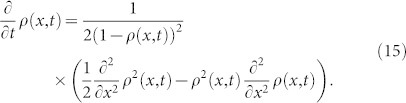
Evaluating the asymptotic solution of this nonlinear PDE, after a long-time evolution, *ρ*(*x*, *t*) becomes much less than unity. As the rough approximation in this long-time limit, we have 1 − *ρ* ≈ 1 and *ρ*^2^ ≈ 0, and Eq. (15) is thus well approximated by 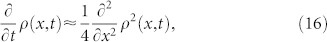
which is nothing but the PME in Eq. (6) with *m* = 2 (*q* = 0). We thus conclude that the approximated asymptotic solution of Eq. (15) is a *q*-Gaussian with *q* = 0. In addition, we can show that this result is mathematically valid by applying the asymptotic Lie symmetry method^51^ (see Method). This method can give an equivalence between the asymptotic solution of the PDE and the analytically-solved one of the other PDE without analytically solving this PDE. Therefore, the associated Markovian model exhibits anomalous diffusion described by the PME in Eq. (6) with *m* = 2. This implies that the interference term of our model leads to the speed-up of the quantum walker *σ_q_*_ = 0.5_ ~ *t*^0.4^ compared to the associated Markovian model *σ_q_*_ = 0_ ~ *t*^1/3^ and makes the zig-zag shape around the *q*-Gaussian distribution.

In summary, we have proposed a feed-forward DTQW model Eq. (4) in which the coin operator depends on the coin states of the nearest-neighbor sites. We show that this model is experimentally feasible. Our feed-forward DTQW model asymptotically satisfies the PME for *m* = 1.5 (*q* = 0.5) and exhibits anomalous slow diffusion *σ_q_*_ = 0.5_(*t*) ~ *t*^0.4^ from the probability distribution and the time dependency of the standard deviation defined in the *q*-Gaussian distribution.

## Discussion

**Figure 4:**
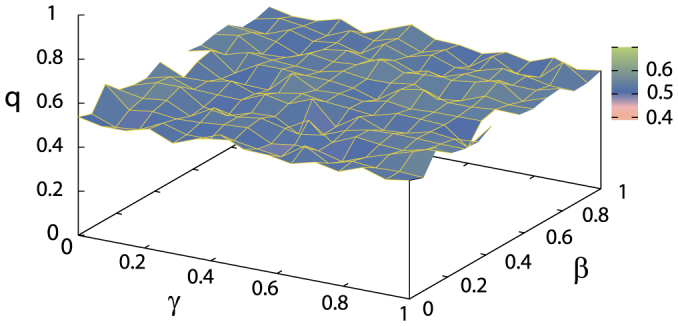
Initial coin state dependence. Changing the parameters *β* and *γ*, we numerically evaluate the parameter *q* of *q*-Gaussian distribution for 225 different initial states expressed by 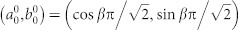 and 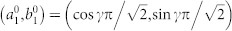. Note that the trivial cases, *β* = 0.5, *γ* = 0 and *β* = 0.5, *γ* = 1, are not plotted. Our fitting result except for the trivial cases is 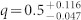.

In this section, we show that our results after the long-time numerical simulations have no initial coin dependence, and that the interference term can be taken as the noise source in addition to the PME. First, while the above analysis uses the only fixed initial coin states as 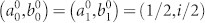, we numerically confirm that there is almost no dependence of the initial coin state except for the trivial cases as follows. We have performed the several numerical simulations for the initial state specified by 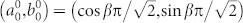 and 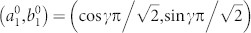 with the real-parameter *β* and *γ* ranging from 0 to 1. Note that the trivial cases, *β* = 0.5, *γ* = 0 and *β* = 0, *γ* = 0.5, lead to the localization of the probability distribution for any time, and we cannot define the parameter *q* for the trivial initial states. Figure 4 shows the numerical evaluation of the parameter *q* of *q*-Gaussian distribution from the data at the two different time steps *t* = 10^6^ and *t* = 10^7^, under the assumption to satisfy the stationary solution of the PME [Eqs. (7) and (8)]. The evaluated *q*-parameters for the various initial states are 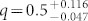 except for the trivial cases. Therefore, we can conclude that our nonlinear model shows the anomalous slow diffusion to satisfy the PME with 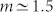 (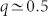) without the initial state dependence.

**Figure 5:**
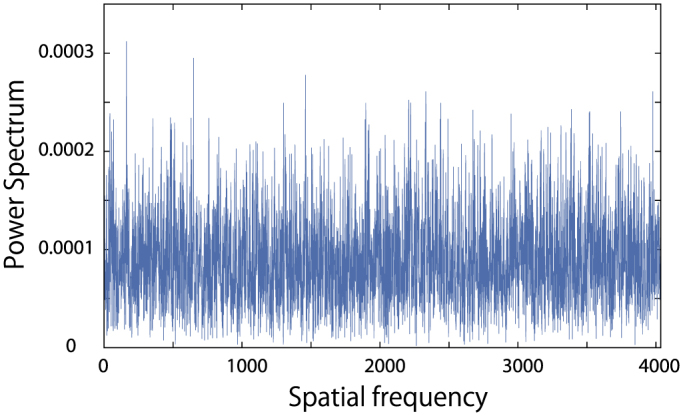
The difference between the nonlinear model and the fit. The power spectrum of the difference between the probability distribution of our model and the *q*-Gaussian with *q* = 0.5 at 10^7^ step. To remove the effects of the expectation value, we replace *x* with *x* − 36.91 in the *q*-Gaussian with *q* = 0.5 [Eq. (7)].

Finally, let us consider the difference between the probability distribution of our model and the *q*-Gaussian distribution with *q* = 0.5, as shown in Fig. 2 (B); the power spectrum of this difference exhibits a white noise as shown in Fig. 5. This power spectrum divided by the physical time scale *t*^0.4^ may remain finite in the asymptotic case, which suggests that our nonlinear model may be mapped to the stochastic PME, i.e. the PME plus a white noise term, in the continuous limit. This stochasticity must come from the interference term. The problem of extracting the stochasticity from a deterministic process has been discussed in another context, that of Mori's noise^52^. Further analysis of this model may reveal the origin of the stochasticity. This is interesting as a purely mathematical problem of a stochastic nonlinear partial differential equation and for showing the relationship between the discrete model and its continuous limit.

## Methods

In what follows, the solution of Eq. (15) is asymptotically identical to the solution of Eq. (16). This is mathematically equivalent to showing that the probability distribution 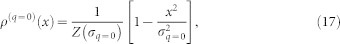
is invariant under an asymptotic Lie symmetry^51^ of the nonlinear partial differential equation (15). In other words, 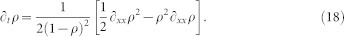
In Eq. (17), *Z*(*σ_q_*_ = 0_) = 4*σ_q_*_ = 0_/3 is the normalization factor, and in what follows, the argument of this function is omitted where possible and ∂*_t_ρ* is denoted as *ρ_t_* for simplicity.

We follow the asymptotic Lie symmetry method and notations in Ref. 51. Under an infinitesimal transformation with the generator 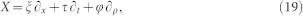
that is 
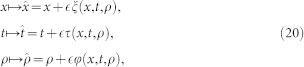
the function *ρ*(*x*, *t*) is mapped to a new function 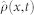, with 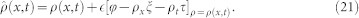
By applying this to the probability distribution Eq. (17), we see that the transformation *X* with *ξ* = −*x* leaves Eq. (17) invariant if and only if 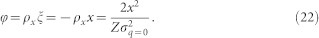
Note that *τ* = *η* · *t* remains unrestricted at this stage because *ρ*^(*q* = 0)^(*x*) does not explicitly depend on time *t*. Conversely, the function *ρ*(*x*) is invariant under 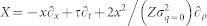 for any *τ* if and only if *ρ*(*x*) is of the form given in Eq. (17).

Following the general procedure for a Lie group analysis of differential equations^53^, the second prolongation of *X* is described by 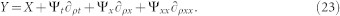
The coefficients Ψ*_t_*, Ψ*_x_*, and Ψ*_xx_* are defined as follows. Under an infinitesimal transformation of *X*, the partial derivatives are transformed as 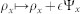, 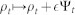, and 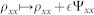. We then readily obtain 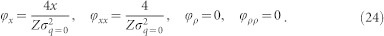
The coefficients Ψ*^t^*, Ψ*^x^*, and Ψ*^xx^* are then obtained by applying the prolongation formula (2.39) from Ref. 52: 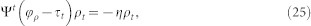


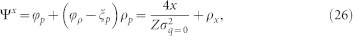


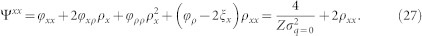
We note that Eq. (18) can be written as 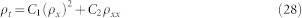
with 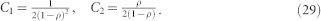
The asymptotic Lie symmetry condition 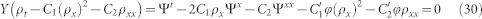
with 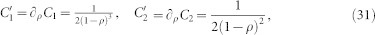
can be written in the following compact form: 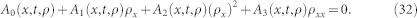
When the condition in Eq. (30) is fulfilled, each *A_k_*(*k* = 0, 1, 2, 3) function must vanish separately in the asymptotic limit 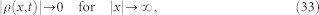
implying that the variance *σ_q_*_ = 0_ also becomes infinity in the asymptotic limit from Eq. (17); 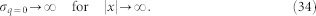
The function *A*_3_ can be expressed as 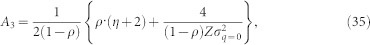
which must be nonzero as *σ_q_*_ = 0_ → ∞, unless we choose 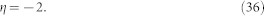
Making this choice, *X* becomes 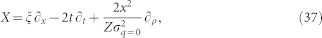
and *A*_3_ reduces to 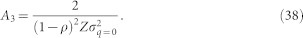
Thus, *A*_3_ → 0 as *σ_q_*_ = 0_ → ∞.

In a similar manner, *A*_0_, *A*_1_, and *A*_2_ are given by 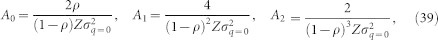
and all become zero as *σ_q_*_ = 0_ → ∞. Therefore, we conclude that the distribution in Eq. (17) is an invariant solution for the transformation *X* of Eq. (37), which is an asymptotic symmetry for large |*x*| of the nonlinear partial differential equation Eq. (18).

## Author Contributions

Y.S. provided the theoretical model of the discrete-time quantum walk; T.W. and J.H. provided the numerical analysis; and T.W. provided the analytical solution of the associated Markovian model with assistance from Y.S. Y.S. conducted this project. All authors contributed to writing the manuscript.

## Supplementary Material

Supplementary Information Probability Distribution of Feed-forward DTQW
