# Supplementary figures and images for: Discrete-time quantum walk with feed-forward quantum coin
Source: Sci Rep. 2014 Mar 21;4:4427. doi: 10.1038/srep04427 (PMC3961739; doi:10.1038/srep04427)

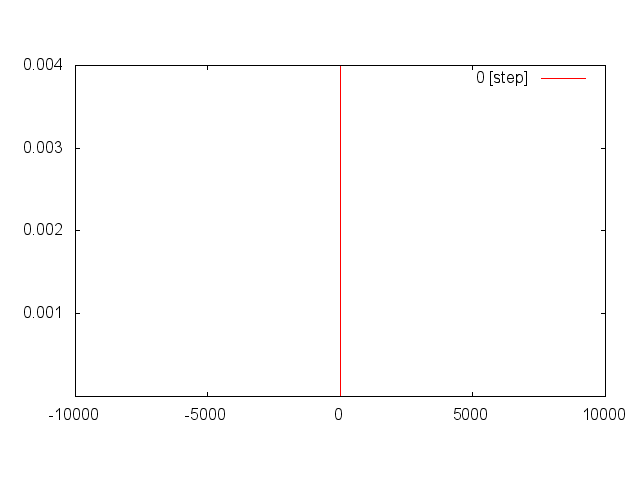

Supplement: Supplementary Information — Probability Distribution of Feed-forward DTQW [file srep04427-s1.gif]
